# The clinical effect of gratitude extension-construction theory nursing program on bladder cancer patients with fear of cancer recurrence

**DOI:** 10.3389/fonc.2024.1364702

**Published:** 2024-04-30

**Authors:** Liping Qian, Yin Zhang, Hui Chen, Yuan Pang, Chenchen Wang, Liangmei Wang, Xiaoqing Zhang

**Affiliations:** Department of Urology, Nanjing Drum Tower Hospital, Affiliated Hospital of Medical School, Nanjing University, Nanjing, Jiangsu, China

**Keywords:** anxiety, cancer, bladder, fear, depression

## Abstract

**Objective:**

To explore the clinical effect of bladder cancer patients with Fear of Cancer Recurrence (FCR) after applying the gratitude extension construction theory nursing program.

**Methods:**

168 patients with bladder cancer hospitalized in the Department of Urology from December 2021 to June 2023 in a hospital are study subjects. The experimental subjects are uniformly designed as an experimental group and a control group, with 52 participants in each group. The former receives routine nursing care, while the later receives nursing interventions based on gratitude extension construction theory. The baseline data, Quality of life Questionnaire-core 30, Quality of Life Questionnaire-non Invasive Bladder Cancer 24, Fear of Progression Questionnaire-Short Form, gratitude level questionnaire, Self-Rating Depression Scale, Self-rating Anxiety Scale, patient compliance behavior score, Overall Survival, and Progression-free Survival are evaluated.

**Results:**

The basic data revealed no statistical significance. The quality of life questionnaire-core 30 and quality of life questionnaire-noninvasive bladder cancer 24 was no significant difference before treatment and after treatment for 1 month. After 9 months, There was a significant difference in pre-treatment scores. The experimental group had no significant difference before and after treatment. For the overall survival rates, the two groups were 67.25% and 79.56%. The progression-free survival rates were 56.35% and 72.35%, respectively, with statistical difference. The compliance rates were 86.54% and 98.08%. The compliance rate of the experimental group exceeded the control group. After 3, 6, and 12 months, the gratitude level questionnaire score and the fear of progression questionnaire-short form in the experimental group were improved. After 3, 6, and 12 months, the control group had no statistically significant difference in the gratitude level questionnaire and the fear of progression questionnaire-short form scores. Compared with the control group, the scores on the gratitude level questionnaire and the fear of progression questionnaire-short form were significantly higher after 3, 6, and 12 months of intervention.

**Conclusion:**

After applying the gratitude extension construction theory nursing program, the FCR of bladder cancer patients is significantly reduced. The quality of life and compliance rate are significantly improved, and anxiety and depression are relieved.

## 1 Background

Bladder cancer is a malignant tumor with high incidence rate in the human urinary system, which has been called the top ten common tumors in the human body. According to the latest research report of the World Health Organization, there are 80000 new cases of bladder cancer in China in 2022. As a tumor with high incidence rate in the urinary system, it directly threatens the life safety of patients (1, 2). Bladder cancer has muscle and non-muscle invasive bladder cancer according to the location. Among them, non-muscle invasive bladder cancer accounts for about 80% of new cases. The gold standard of clinical medical treatment for non-muscle invasive bladder cancer is transurethral tumor resection. However, this surgical treatment has been proven to have a high recurrence rate. The 5-year recurrence rate is as high as 60%. A few patients progress to muscle invasive bladder cancer. After regular bladder infusion, patients still exhibit a high tumor recurrence rate, with a 5-year survival rate below 50%, and even worse (3, 4). Therefore, bladder cancer patients are prone to Fear of cancer recurrence (FCR), which is also the most frequent and strongest psychological problem of cancer patients. In clinical work, it is far from enough to only focus on the platform therapy of cancer. The psychological status of cancer patients should be paid more attention. Identifying high FCR and formulating effective intervention measures are crucial for the prognosis of bladder cancer patients. The gratitude extension construction theory and cognitive-behavioral therapy, as research areas in psychology, are gradually being valued for their clinical value in diseases such as cancer. Appropriate FCR will contribute to recovery and actively cooperate with clinical doctors in their treatment plans. However, excessive FCR will lead to severe anxiety, depression, and other negative psychological problems in patients, thereby reducing treatment compliance, increasing the incidence of chemotherapy drugs, and ultimately resulting in the inability to achieve the expected treatment effect. FCR is positively correlated with negative emotions such as anxiety and depression. Cancer patients with higher negative emotions will spend more time collecting disease-related information. Some erroneous information may exacerbate their fear of recurrence, forming a vicious cycle that will greatly affect their physical and mental health and quality of life.

Fredrickson point out that from the perspective of extension and construction, gratitude, as a positive emotional state, can be intervened and measured with low investment costs and simple intervention methods. It can not only expand temporary thinking action plans, but also help individuals construct advanced cognitive and social resources, effectively correcting, repairing, and mitigating the adverse consequences of negative emotions, and improving individual adaptability and life satisfaction (5, 6). The effectiveness of the program has been confirmed in other diseases such as cervical cancer, oral cancer, breast cancer, etc. To deeply analyze the therapeutic effect of this theory in bladder cancer FCR and verify the effectiveness of psychological research theory, the study conducted in-depth analysis on this theory, providing a reference for alleviating FCR emotion in patients with bladder cancer. The contributions of the study are as follows. Nursing intervention based on gratitude extension construction theory can effectively reduce the FCR of bladder cancer patients, alleviate anxiety and depression, and improve their gratitude level. Bladder cancer patients have moderate fear for cancer recurrence. The FCR of bladder cancer patients is affected by many factors. Interventions should be strengthened for young, highly educated, economically burdened, anxious and depressed patients, as well as weak social support systems. The research structure is as follows. In the first part, data is collected and grouped. Different groups adopt different intervention methods. The intervention results are observed through a survey questionnaire. The second part describes the research results and analyzes whether there are differences in the data through statistical methods. The third part analyzes the research results. The research results of other scholars are presented to support them, while also supplementing the shortcomings of the research.

## 2 Theoretical framework

The gratitude extension construction theory, as a new type of psychological theory, allows individuals with high gratitude to express more positive emotions, shift their focus from negative events to experiencing positive things, and thus stay away from negative emotions and experiences. The gratitude extension construction theory proposes that each emotion has a unique evolutionary purpose and adaptability. Positive emotions expand attention, cognition, and action, and establish multiple available resources to eliminate physical discomfort caused by negative emotions. The gratitude extension construction theory has an expanding function. The gratitude extension construction theory suggests that gratitude, as an emotional trait, has a priming and expanding effect on cognition. It can not only induce positive emotions to broaden people’s attention and thinking activities, but also expand individuals’ cognitive abilities and promote their attention and processing of important information. The gratitude extension construction theory has a constructive function. Gratitude can construct positive psychological capital on the basis of “expansion”, help individuals construct sustainable resources in various aspects, improve psychological adaptability, and increase happiness. Gratitude intervention, whether it is a private gratitude diary or a public expression of gratitude, can enhance a person’s sense of gratitude towards others, enable them to better handle relationships with others, and improve their social adaptability. The gratitude extension construction theory has a slow-release function. The gratitude extension construction theory suggests that positive emotions can change people’s thinking and behavior, and regulate negative emotions. Gratitude has the functions of correction, repair, and slow-release, which can correct and eliminate adverse reactions of negative emotions, shorten the time for awakening negative emotions, and delay the progression of diseases.

## 3 Methods

### 3.1 General information

168 patients with bladder cancer hospitalized in the Department of Urology from December 2021 to June 2023 are selected as study subjects. They are divided into the Control Group (CG) and the Experimental Group (EG), with 52 cases in each group. Inclusion criteria: ① The patient conforms to the relevant diagnostic principle of non-muscle invasive bladder cancer. The patient is diagnosed by pathology. ② The clinical pathological staging is stage I. ③ The survival time shall not be less than half a year. Exclusion criteria: ① Patients who are allergic to chemotherapy drugs or immune drugs. ② Menopausal women. ③ The patient has other serious illnesses. ④ The patient is simultaneously subjected to radiation therapy. The study is approved by the hospital committee. Patients voluntarily participate in clinical research. The calculation formula for sample size is the main observation indicator for comparing the means of two samples, as shown in Equation (1).


(1) $$n_{1}=n_{2}=2\times {\left[\left(\mu_{\alpha }+\mu_{\beta }\right)\sigma /\delta \right]}^{2}$$

In Equation (1), $n_{1}$ and $n_{2}$ are different samples. $\mu_{\alpha }$ and $\mu_{\beta }$ are the mean of the samples. σ and δ are the standard deviation and difference of different samples. α=0.05 , and β=0.1 . According to the bilateral test and related research settings, considering a 20% dropout rate, the final sample size included in the study is 168 cases, with 52 cases in each group.

### 3.2 Research method

The research design type is retrospective research. Research objects are invited to participate in the research on the effectiveness of the gratitude extension construction intervention theory in cancer patients with FCR. The purpose of this research is to explore whether gratitude intervention will have a positive impact on FCR, anxiety, depression and gratitude of bladder cancer patients. This research lasts for about three months, which is conducive to medical staff evaluating FCR status and formulating the best intervention measures. This will further improve the quality of medical care and also help patients recover as soon as possible. The research subjects are invited to fill out questionnaires. This questionnaire does not involve any privacy, and the answers are correct or incorrect. Please do not have any concerns. If this study incurs a time burden, one can freely choose whether to participate or withdraw from the study at any time. All information related to research is confidential. The research subjects are free to choose to participate or refuse to participate, and have the right to withdraw from this study at any time during the research process. The CG receives routine nursing care, including admission education, psychological care, postoperative guidance, health education, bladder infusion chemotherapy regimen, and bladder infusion related nursing. Admission education refers to warmly receiving patients and establishing a good nurse patient relationship of mutual trust with them. Nursing is carried out according to the routine nursing procedures of urology department. Bladder cancer health education manuals are distributed to them to eliminate their nervousness about strange environment. Postoperative guidance involves oral education to inform patients of postoperative precautions, common adverse reactions and coping strategies during postoperative chemotherapy, and the importance of timely infusion chemotherapy and regular follow-up. Health education is to guide patients to quit smoking, avoid contact with harmful substances such as rubber, plastics, dyes, etc., exercise properly, strengthen physique, and reduce the recurrence rate of bladder cancer. Psychological nursing only includes doctors and nurses introducing the treatment effect and prognosis of bladder cancer to patients, so as to reduce their fears. Successful cases are used to encourage patients and help them build confidence in overcoming the disease. The bladder infusion chemotherapy regimen is performed after surgery, once a week, lasting 8 consecutive times, then once a month, lasting 1-2 years.

**Figure 1:**
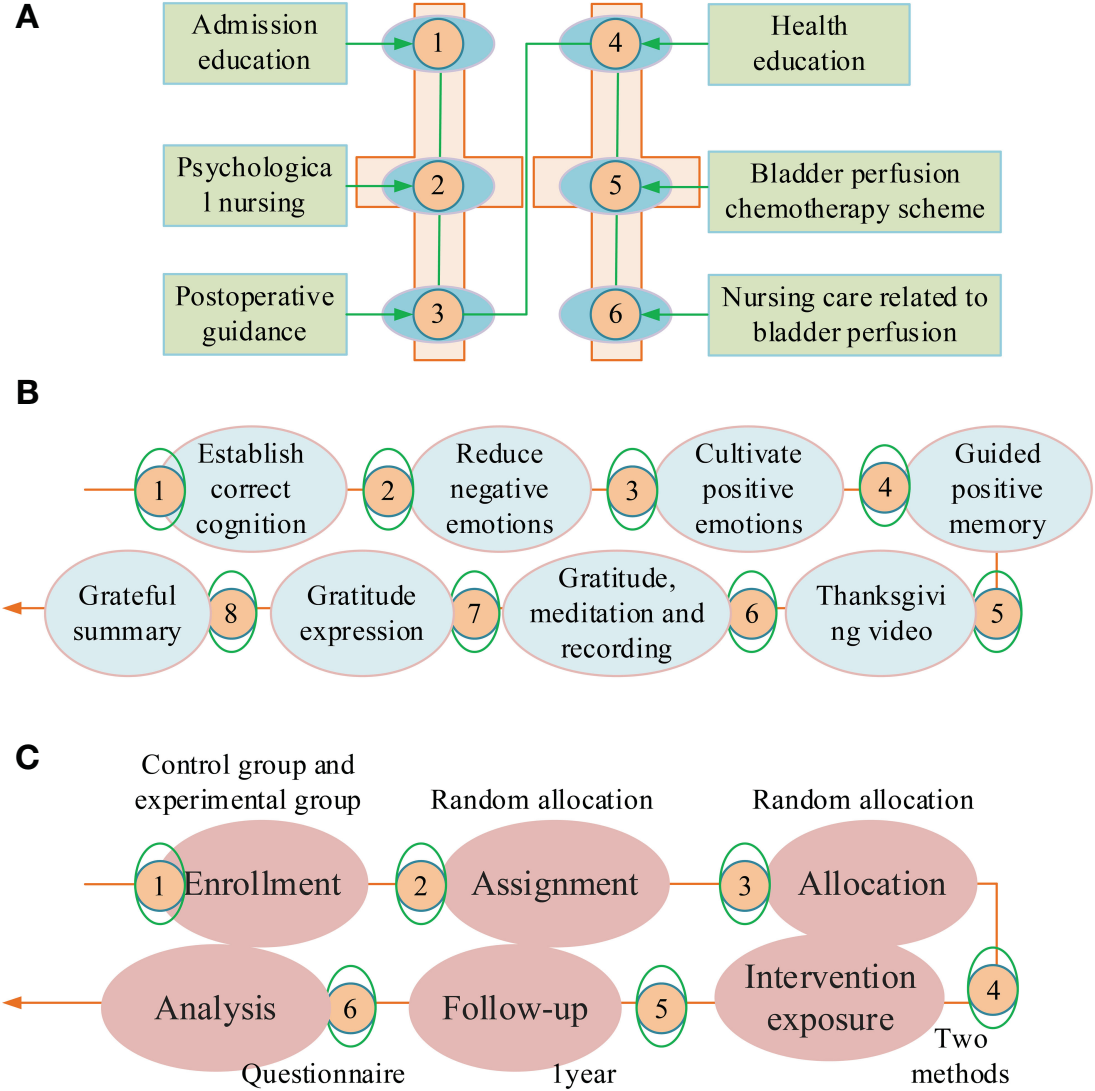
Different nursing methods adopted by two groups of patients. **(A)** Nursing methods of control group. **(B)** Nursing methods of experimental group. **(C)** Proceess diagram of participants in the study.

On the basis of conventional nursing methods, the EG adds gratitude extension construction theory to complete nursing intervention, each time for about 20-30 minutes, once a week, for a duration of 8 months. To understand the research progress of cancer recurrence fear at home and abroad in the past 10 years and the related content of gratitude extension construction theory, and combined with the characteristics of cancer, all researchers jointly explore to develop a preliminary nursing intervention plan based on the gratitude extension construction theory. The time, form, and content of intervention are determined based on the psychological characteristics of patients and the problems encountered in clinical nursing practice. To ensure the smooth progress of the later experiment and make researchers more familiar with the intervention methods and procedures, a 4-week pre-survey is conducted on 5 patients before the official start of the experiment. Based on the problems found in the pre-experiment, the intervention plan is adjusted and improved timely. To ensure the effectiveness of psychological intervention programs based on gratitude extension construction theory, research needs to be evaluated by an expert team. The expert team consists of one nursing expert, three psychology experts, one urology nursing expert, and one medical specialist. The work experience exceeds 15 years. The psychological intervention program based on the gratitude extension construction theory is divided into 8 stages, including establishing correct cognition, reducing negative emotions, cultivating positive emotions, guiding positive memory, gratitude videos, gratitude contemplation and recording, gratitude expression, and gratitude summary. The specific implementation content for each stage is as follows. For establishing correct cognition, after the patient is admitted, the implementer participates in ward rounds with the doctor, introduces themselves with the doctor, and communicates with the patient. Ask patients about their understanding of bladder cancer and their psychological state after diagnosis. Answer the patient’s questions, provide them with correct understanding, and introduce some successful treatment cases. For reducing negative emotions, encourage patients to express their fears and fearful emotions. Simultaneously, guide them to analyze the different impacts of excessive and moderate FCR on health status. Analyze the clinical value of alleviating excessive FCR. Guide patients to actively face FCR. For cultivating positive emotions, apply positive psychology related statements to comfort patients, and guide them to establish a positive attitude. Use positive words in communication with patients. During infusion chemotherapy, cultivate their hobbies such as flower cultivation, listening to music, playing chess, etc., to alleviate the discomfort caused by infusion chemotherapy. For guiding positive memory, guide patients to recall happy memories in their daily lives and describe their living conditions through positive language. Encourage patients to spend more time with their families completing daily tasks. For gratitude videos, collect and edit video collections to guide patients in watching, including videos such as “Gratitude for Changes in Mental Life” “Gratitude for Cancer” and “Gratitude for Parents”. For gratitude contemplation and gratitude recording, during bladder infusion therapy, guide patients to think about whether there have been grateful people and things in the past few weeks. Guide patients to recall things they are grateful for within ten minutes, while gradually shifting their focus to things that they are grateful for, recalling the scene and feelings at that time. After completing the infusion process, cooperate with the patient to record the content in the form of gratitude and contemplation. The implementer can view the gratitude record and analyze the problem accordingly. For the gratitude expression stage, guide patients to express gratitude to their loved ones and friends who provided assistance during their illness. The ways to express gratitude are verbal expression, blessing cards, phone calls, and text messages. For the gratitude summary stage, encourage patients to share the positive emotional experiences and gains brought about by the determination of gratitude emotions. Drive patients to recall the intervention content of the first seven stages, organize relevant suggestions, and summarize the entire intervention process. **Figures 1A, B** refer to the different nursing methods used in two groups. **Figure 1C** refers to the process of participants at each stage, including enrollment, assignment, allocation, and intervention exposure, follow-up, and analysis.

### 3.3 Observed indicator

The patients are evaluated based on clinical baseline data, Quality of life Questionnaire-core 30 (QLQ-C30), Quality of Life Questionnaire-non Invasive Bladder Cancer 24 (QLQ-BLS24), Fear of Progression Questionnaire-Short Form (FoP-Q-SF), gratitude level questionnaire, Self-Rating Depression Scale (SDS), Self-rating Anxiety Scale (SAS), patient compliance behavior score, Overall Survival (OS), and Progression-free Survival (DFS). FoP-Q-SF is the primary observation indicator, while the remaining indicators are secondary observation indicators. The survey questionnaire used has been calculated to have extremely high reliability and validity, with a reliability value exceeding 0.78 and a validity value exceeding 0.82. In addition frequent information, basic data includes tumor diameter, pathological type and Tumor-Node-Metastasis (TNM) stage. QLQ-C30 has 3 scales, 5 fields, and 30 items. The 3 scales are symptom, function, and overall health. The five fields are emotion, body, society, cognition, and role. QLQ-BLS24 includes three symptom sub-scales and two functional sub-scales. Three symptom sub-scales include treatment related symptoms, intestinal symptoms, and urinary tract symptoms, while two functional sub-scales include concerns about the future and sexual function. A high score on the symptom sub-scale indicates significant symptoms. The quality of life for patients is even worse. If the functional sub-scale score is high, it indicates that the patient’s functional status is better. FoP-Q-SF includes two dimensions: social family and physical health, each with 6 items, totaling 60 points. The scale is scored using the Likert 5-point rating method. The scoring ranges for low fear, moderate fear, and high fear are 12-23 points, 24-36 points, and 37-60 points, respectively. The gratitude level questionnaire includes 6 questions. The Likert 7 scoring method is adopted, namely strongly disagree-strongly agree. Questions 3 and 6 are reverse scoring questions. A higher score indicates higher gratitude. The compliance behavior includes activity, examinations, medication, diet, and emotions. 0-1 item is non-compliant. 2-4 items are partial compliant. Adhering to all items is a complete medical compliance behavior. Complete compliance with medical treatment and partial compliance with medical treatment are considered compliance rates. After treatment, the analysis is conducted at two stages, 3 and 9 months, with follow-up period for 1 years. The observation time for the gratitude survey questionnaire and FoP-Q-SF is set to 3, 9, and 12 months.

### 3.4 Statistical methods

Epidata3.0 software conducts two-way data analysis. The SPSS 23.0 software is applied to data analysis. Due to the fact that the data consists of quantitative and qualitative data, as well as different data from the same sample in a time series, different statistical testing methods are used in the experiment based on the data feature. The χ^2^ is used for inter group test. The mean ± standard deviation ( $\bar{\text{x}}$ ± s) is used to describe metrological data that conforms to a normal distribution. The comparison between two groups is conducted through a test. The comparison analysis at different time is conducted using the variance repeat test. Non-parametric detection is applied to inter group difference analysis. The Kruskal-Wallis is applied to compare data. The *t*-test is used to compare whether the difference between two means is statistically significant. The variance repeat test is a *post hoc* test method that mainly focuses on the differences between the dependent variables at different time or conditions. Non parametric detection is used to infer population distribution patterns using sample data when the population variance is unknown or poorly understood. The Kruskal Wallis test is a non-parametric test used to compare groups of two or more continuous or discrete variables. The baseline data for equivalence in the research group is 0.135. The statistical method for baseline differences is standardized analysis. The Kaplan-Meier and logarithmic rank test calculate the OS and DFS. The Bland-Altman analysis chart is used to analyze the consistency. *P*<0.05 indicates statistical significance. *P*<0.01 indicates significant statistical significance.

## 4 Results

### 4.1 Comparison of general clinical data

**Table 1** is the baseline data of the two groups. There was comparability in basic data such as gender, age, clinical stage, education level, tumor histological grading, and chemotherapy drugs between the two groups, (*P*>0.05).

**Table 1:** Comparison of baseline data [(x ± s)(n/%)].

Classification		Control group N=52	Experiment Group N=52	χ2/*t* value	*P* value
Sexual distinction	Male	40/76.92	42/80.77	0.621	0.884
	Female	12/23.08	10/19.23		
Age/Year		46.32 ± 10.34	48.82 ± 9.65	0.784	0.512
Tumor staging	III	29/55.77	27/51.92	0.625	0.887
	IV	23/44.23	25/48.08		
Degree of education	Primary and junior high school	14/26.92	15/28.85	0.725	0.824
	High school	12/23.08	13/25.00		
	Junior college	10/19.23	9/17.31		
	Undergraduate	16/30.77	15/28.85		
Tumor histological grading	Low level	28/53.85	29/55.77	0.681	0.878
	High level	24/46.15	23/44.23		
Perfusion medication	Epirubicin	19/36.54	18/34.62	0.725	0.456
	Pirarubicin	20/38.46	18/34.62		
	Other	13/25.00	14/26.92		

### 4.2 Comparison of QLQ-C30

The QLQ-C30 scores are shown in **Table 2**. For the two groups, the QLQ-C30 scores had no significant difference before therapy and one month after treatment, (*P*>0.05). After 9 months, the QLQ-C30 score had significant difference, (*P*<0.001). Before and after treatment, the EG had significant significance, (*P*<0.05).

**Table 2:** QLQ-C30 for two groups.

Group		Control group	Experiment Group	Cohen’s d	95%*CI*	*t*	*P*
Emotional function	Before intervention	53.68 ± 7.08	56.35 ± 7.02	0.23	[49.67,58.24]	1.105	0.271
	After 3 month of intervention	58.67 ± 6.54	60.23 ± 4.56	0.29	[54.39,62.37]	0.478	0.634
	After 9 months of intervention	64.57 ± 8.05	71.64 ± 9.57	0.31	[59.36,69.15]	3.808	<0.001
Cognitive function	Before intervention	58.20 ± 7.13	57.06 ± 6.94	0.32	[52.36,62.35]	0.819	0.415
	After 3 month	62.42 ± 7.86	65.32 ± 6.85	0.78	[58.18,65.19]	1.80	0.076
	After 9 months	65.11 ± 8.05	72.04 ± 9.26	0.22	[59.59,70.16]	4.073	<0.001
Role Functions	Before intervention	56.44 ± 5.22	55.74 ± 6.82	0.19	[51.26,60.68]	0.922	0.359
	After 3 month	60.15 ± 5.74	63.48 ± 5.37	0.38	[58.25,63.33]	1.708	0.089
	After 9 months	66.15 ± 8.43	73.46 ± 10.15	0.28	[59.17,73.26]	3.957	<0.001
Somatic function	Before intervention	56.05 ± 7.35	57.13 ± 6.49	0.16	[50.37,62.29]	0.792	0.424
	After 3 month of intervention	61.25 ± 7.89	64.23 ± 8.35	0.54	[57.21,67.27]	1.637	0.102
	After 9 months of intervention	66.43 ± 8.16	78.04 ± 10.26	0.18	[55.26,71.66]	6.386	<0.001
Social function	Before intervention	57.19 ± 6.02	55.94 ± 6.75	0.29	[52.05,65.36]	0.986	0.321
	After 3 month of intervention	62.32 ± 6.58	63.52 ± 7.58	0.75	[55.56,64.36]	1.347	0.148
	After 9 months	63.86 ± 7.53	74.52 ± 9.85	0.15	[29.25,37.26]	5.037	<0.001
Overall health	Before intervention	50.17 ± 11.57	52.68 ± 9.65	0.18	[43.34,58.25]	0.986	0.308
	After 3 month	57.27 ± 9.86	59.09 ± 10.68	0.46	[50.38,65.87]	1.257	0.208
	After 9 months	59.28 ± 10.85	67.58 ± 13.08	0.28	[49,39.68.25]	6.231	<0.001

### 4.3 Comparison of QLQ-BLS24

The QLQ-BLS24 scores are shown in **Table 3**. There was no significant difference in the QLQ-BLS24 sub-scale scores between the two groups before and one month after therapy, (*P*>0.05). After 9 months, the QLQ-BLS24 scores had significant differences, (*P*<0.001). Before and after treatment, the experiment group had significant differences, (*P*<0.05).

**Table 3:** QLQ-BLS24 scores for two groups.

Group		Control group	Experiment Group	Cohen’s d	95%*CI*	*t*	*P*
Treatment related symptoms	Before intervention	35.62 ± 5.36	34.25 ± 4.68	0.15	[29.25,37.26]	1.105	0.269
	After 3 month of intervention	29.35 ± 7.96	29.53 ± 7.56	0.19	[23.06,34.86]	0.467	0.623
	After 9 months of intervention	23.63 ± 8.88	24.35 ± 9.36	0.46	[16.38,29.87]	3.735	<0.001
Intestinal symptoms	Before intervention	35.66 ± 7.13	34.06 ± 6.94	0.29	[22.36,39.62]	0.758	0.415
	After 3 month of intervention	28.91 ± 7.86	28.32 ± 6.85	0.35	[21.05,34.98]	1.756	0.076
	After 9 months of intervention	23.65 ± 8.05	23.47 ± 9.32	0.73	[17.56,28.36]	5.236	<0.001
Urinary tract symptoms	Before intervention	36.36 ± 6.55	35.36 ± 7.18	0.25	[29.59,40.36]	0.920	0.354
	After 3 month of intervention	28.63 ± 6.35	29.36 ± 6.68	0.16	[21.26,34.68]	1.688	0.088
	After 9 months of intervention	23.65 ± 7.89	23.56 ± 7.88	0.43	[18.22,28.33]	3.978	<0.001
Worries about the future	Before intervention	58.36 ± 6.59	57.25 ± 7.25	0.20	[29.25,37.26]	0.986	0.321
	After 3 month of intervention	68.25 ± 7.28	75.25 ± 8.36	0.18	[22.36,35.26]	1.256	0.086
	After 9 months of intervention	72.56 ± 8.68	85.69 ± 10.35	0.55	[64.25,79.25]	5.037	<0.001
Sexual function	Before intervention	60.17 ± 11.57	62.68 ± 9.58	0.19	[49.26,71.26]	0.986	0.308
	After 3 month of intervention	67.27 ± 9.86	72.35 ± 10.56	0.26	[57.05,65.36]	1.257	0.208
	After 9 months of intervention	72.28 ± 10.79	82.56 ± 14.62	0.78	[60.56,80.36]	6.231	<0.001

### 4.4 Comparison of SDS and SAS between two groups

**Figure 2:**
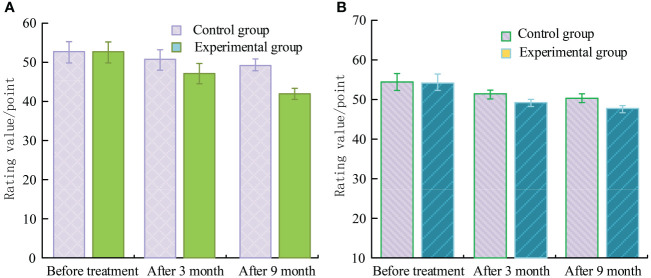
SDS and SAS scores. **(A)** SAS rating. **(B)** SDS rating.

The SDS and SAS are displayed in **Figures 2A, B**, respectively. Before therapy, there was no variation in their scores, (*P*>0.05). After 9 months, they had significant differences, (*P*<0.001). Before and after treatment, there was a significant difference in the EG, (*P*<0.05).

### 4.5 Evaluation of compliance behavior between two groups

**Table 4** shows the compliance behaviors. The patients in the CG and EG who fully complied with medical treatment were 22 and 34, respectively. The partially compliant patients were 23 and 17, respectively. The compliance rates of the CG and the EG were 86.54% and 98.08%, respectively. The compliance rate of the EG exceeded the CG, (*P*<0.05).

**Table 4:** Compliance behavior assessment.

Group	Control group	Experiment Group	χ2	*P*
Complete compliance	22 (42.31)	34 (65.38)	3.768	0.047
Partial compliance	23 (45.10)	17 (32.69)	0.365	0.545
Non compliance	7 (13.46)	1 (1.92)	5.857	0.013
Compliance rate	44 (86.54)	51 (98.08)	4.251	0.034

### 4.6 Comparison of survival curves between two groups

**Figure 3:**
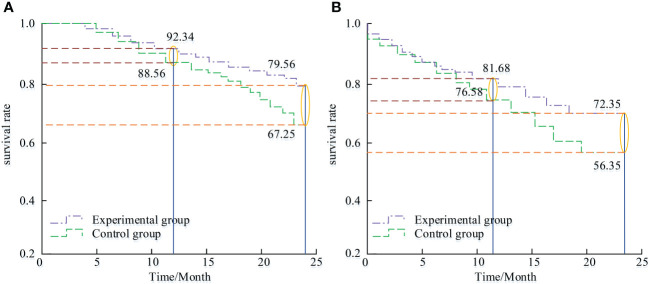
OS curve and DFS curve of two groups of patients. **(A)** OS. **(B)** PFS.

**Figure 3** was the OS and DFS curves of the two groups, respectively. Overall, from the 2-year follow-up, the OS of the CG and the EG were 67.25% and 79.56%, respectively. The DFS of the two groups were 56.35% and 72.35%, respectively, (*P*<0.001).

### 4.7 Comparison of gratitude level questionnaire scores between two groups

**Figure 4:**
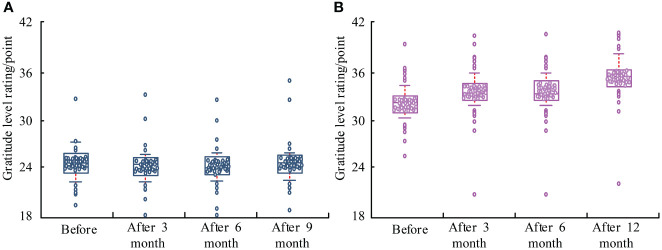
Comparison of gratitude level questionnaire scores. **(A)** Control group. **(B)** Experimental group.

**Figure 4** compares the scores of the gratitude level questionnaire. After 3, 6, and 12 months, the gratitude level questionnaire scores of the EG was improved, (*P*<0.05). After 3, 6, and 12 months, the CG had no significant change, (*P*>0.05). The gratitude level questionnaire scores of EG was improved obviously, (*P*<0.05).

### 4.8 Comparison of FoP-Q-SF scores between two groups

**Figure 5:**
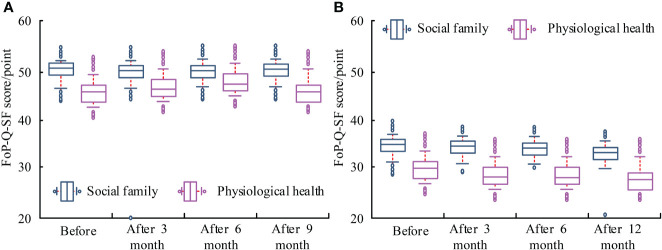
Comparison of FoP-Q-SF scores between two groups of patients. **(A)** Control group. **(B)** Experimental group.

Before treatment, the score difference was not significant, (*P*>0.05). After 3, 6, and 12 months, there was a significant change in the rating, (*P*<0.001). Before and after treatment, the scores of the EG were significant, (*P*<0.05). The results are displayed in **Figure 5**.

### 4.9 Bland-Altman analysis chart results

**Figure 6:**
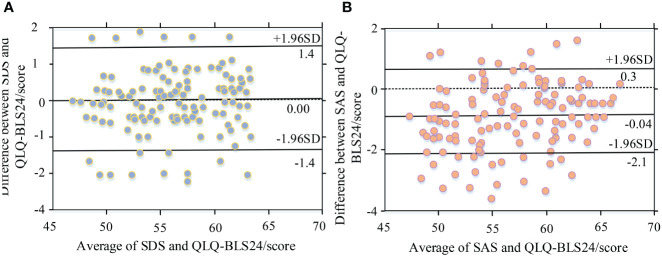
Blank Altman analysis of SDS and SAS withQLQ-BLS24. **(A)** Blank Altman analysis chart for SDS abd QLQ-BLS24. **(B)** Blank Altman analysis chart for SAS and QLQ-BLS24.

**Figure 6A** shows the Bland-Altman results for SDS and QLQ-BLS24. **Figure 6B** shows the Bland-Altman for SAS and QLQ-BLS24. The mean was shown by the horizontal axis. The difference was along the vertical axis. For the SDS and QLQ-BLS24, the mean was 0. The upper and lower limits of the 95% confidence interval were 1.4 and -1.4. The proportions of outliers were 3.23% and 5.38%, respectively. The consistency limits had a maximum difference of 0.81 and 0.63, respectively. Regarding the QLQ-BLS24 results, SAS and SDS demonstrated strong consistency.

## 5 Discussion

With the progress in medical nursing models, the impact of psychological care for cancer patients on treatment is gradually being emphasized. This can not only improve the mental health of patients, but also to some extent improve their quality of life. Gratitude extension construction theory is the most successful psychological intervention program at present, which has positive therapeutic effects in breast cancer, uterine cancer, liver cancer, lung cancer and other cancers. The functions of gratitude extension construction theory include expansion function, construction function, and slow-release function. The intervention strategies of gratitude extension construction theory include four aspects: gratitude diary, gratitude contemplation, gratitude expression, and gratitude video. These strategies are not only a good response to the kindness of others, but also a positive force with social morality. The basic data of the two groups are comparable. The difference is not statistically significant. Therefore, the subsequent research results have reference value.

According to the research, there was no significant difference in the QLQ-C30 and QLQ-BLS24 scores between the two groups before and one month after treatment. The EG showed a significant difference before and after treatment. This may be because gratitude intervention can stimulate the prefrontal cortex of the brain, which can alleviate the harm of negative emotions to physical and mental health. Gratitude can activate and expand personal cognition. This not only broadens thinking activities and attention range, but also enhances the ability to pay attention to and process important information. Gratitude can establish positive psychological capital, establish sustainable resources in multiple fields, enhance psychological adaptability, and improve happiness experiences. Gratitude can adjust negative emotions timely, reduce the response time of negative emotions, and thus alleviate the condition. Volz Y et al. proposed that gratitude records could alleviate patients’ anxiety and stimulate their conscious initiative. Gratitude meditation could express the patient’s gratitude, improve their emotional management ability, and promote their physical and mental health (7, 8). Rammant E et al. proposed that positive memory and hope, gratitude records, gratitude expression and other psychological interventions could reduce the psychological pain of diabetes patients, effectively control blood sugar, and improve quality of life (9, 10). Appleyard S E suggested that gratitude intervention could stimulate patients to recall positive gratitude events, further excavate positive forces around them, and thus improve their gratitude (11). Magnani C et al. pointed out that expressing gratitude and sharing positive experiences stimulated positive emotions such as gratitude, happiness, and optimism in cervical cancer patients, effectively adjusting psychological state and improving overall happiness (12).

FCR is a common psychological issue among cancer survivors. This is also the biggest psychological problem for cancer patients. Research suggested that newly diagnosed cancer patients typically had higher FCR psychological issues. FCR is also an important reason for low patient follow-up compliance. After 3, 6, and 12 months of intervention, they had no difference in the FoP-Q-SF score in the CG. Compared with the EG, the FoP-Q-SF score significantly increased after intervention for 3, 6, and 12 months. This was consistent with the clinical application effect of this theory in other chronic cancers (11, 13, 14). Cary C et al. believed that higher FCR triggered stronger anxiety and depression. The quality of life also decreased accordingly. Meanwhile, the severity of FCR in patients also has changed over time (15, 16). Cancer patients are becoming increasingly aware of their condition. Richards H L et al. believed that FCR gradually decreased, which was consistent with the research findings (17, 18). This may be when facing traumatic events, high-level cognition helps patients rebuild resources destroyed by the crisis. These reserve resources can encourage individuals to effectively respond to negative emotions and other negative events, correct and eliminate adverse reactions to negative emotions.

The overall survival rates of the CG and the EG were 67.25% and 79.56%. The progression-free survival rates were 56.35% and 72.35%, with significant statistical difference. The compliance rates of the CG and the EG were 86.54% and 98.08%, respectively. The compliance rate of the EG exceeded the CG. Meanwhile, research suggested that the scores of SDS and SAS significantly improved after applying the gratitude extension construction theory nursing plan. This may be because an increase in gratitude helps reduce negative emotions towards unknown diseases. After 3, 6, and 12 months of intervention, the gratitude level questionnaire of the EG significantly improved. After 3, 6, and 12 months of intervention, there was no statistically significant difference in the gratitude level questionnaire scores of the CG. Compared with the EG, the gratitude level questionnaire scores were significantly higher after 3, 6, and 12 months of intervention. This is consistent with the clinical application of this theory in other chronic cancers (19, 20). The gratitude level is highly correlated with the molecular level of neuropeptide oxytocin in the inner brain, which can reduce inflammation in the human body, alleviate depression and anxiety in patients, and enhance happiness (21). Volz Y et al. analyzed the predictive factors for high FCR in patients with urogenital cancer through prospective trials. The research results showed that anxiety, depression, and other negative psychological factors in patients increased the probability of FCR in urogenital cancer patients. This is consistent with the research results (22). Tan W S et al. analyzed the experience of patients with non-muscle invasive bladder cancer after diagnosis. The research methods were brief disease cognition questionnaire and semi-structured interview. The test results showed that early and timely transurethral bladder tumor resection and psychological intervention could help reduce patients’ negative emotions (23, 24). The research results have certain limitations, such as account study hypotheses, sources of potential bias, measurement accuracy, multiple analyses, and other limitations or weaknesses of the study. The study only includes bladder cancer patients in a hospital, with a small sample size and insufficient representation. It is suggested to conduct a multi-center, large sample longitudinal study in the future to observe the dynamic changes of FCR in bladder cancer patients, and further analyze the long-term change trajectory and influencing factors of FCR in bladder cancer patients. Due to limitations in manpower and resources, the follow-up time of this study is relatively short. The long-term effects of nursing intervention plans can be further validated in the future.

## 6 Conclusion

Combined with the most advanced research reports and results at home and abroad, the quality of life and compliance rate of bladder cancer patients have significantly improved after implementing the gratitude extension construction theory nursing program. Patients have higher gratitude and significant decrease in FCR, which in turn alleviate anxiety and depression. However, the sample quantity is limited. They are all from tertiary hospitals in the same region. The results may be biased. More studies are required to provide evidence. The main responsibility for the nursing plan of the gratitude extension construction theory adopted is the hospital, without other participants. Further research should be conducted to improve the nursing quality.

## Data availability statement

The original contributions presented in the study are included in the article/supplementary material. Further inquiries can be directed to the corresponding author.

## Ethics statement

The study was approved by the local ethics committee of the Affiliated Hospital of Medical School, Nanjing University. All experiments were performed in accordance with relevant guidelines and regulations such as the Declaration of Helsinki and the patients signed the informed consent form and agreed to be published. The studies were conducted in accordance with the local legislation and institutional requirements. Written informed consent for participation in this study was provided by the participants’ legal guardians/next of kin.

## Author contributions

LQ: Conceptualization, Methodology, Formal analysis, Writing – original draft, Writing – review & editing, Visualization, Investigation, Validation. YZ: Methodology, Formal analysis, Writing – review & editing, Data curation, Investigation, Validation. HC: Formal analysis, Writing – review & editing, Data curation. YP: Writing – review & editing, Data curation. CW: Writing – review & editing, Data curation. LW: Supervision, Project administration, Writing – review & editing. XZ: Supervision, Project administration, Writing – review & editing.
